# Complete Genome Sequence of Porcine Epidemic Diarrhea Virus Strain COL/Cundinamarca/2014 from Colombia

**DOI:** 10.1128/genomeA.00239-16

**Published:** 2016-04-21

**Authors:** Matthew C. Jarvis, Ham Ching Lam, Albert Rovira, Douglas G. Marthaler

**Affiliations:** Department of Veterinary Population Medicine, College of Veterinary Medicine, University of Minnesota, St. Paul, Minnesota, USA

## Abstract

Porcine epidemic diarrhea virus (PEDV) has been found throughout Europe and Asia, and has emerged in North and South America. A whole genome sequence was obtained from a paraffin-embedded tissue sample from the Instituto Colombiano Agropecuario (ICA), Colombia through Next Generation Sequencing techniques to further understand the evolution of PEDV.

## GENOME ANNOUNCEMENT

Porcine epidemic diarrhea virus (PEDV) is a single stranded, positive sense RNA virus that causes dramatic mortality in neonatal piglets. The pathogen was discovered in the United Kingdom in 1971, and was later found throughout Europe and Asia (1, 2). In 2010, a pathogenic PEDV strain emerged in China, and in 2013, two main strains emerged in the Americas, including the pathogenic and S-indel strains, which caused massive financial losses for the United States swine industry (3). In addition, PEDV outbreaks have been reported in Central and South America, including Mexico, Peru, the Dominican Republic, Ecuador, and Colombia (3, 4).

On 2 May 2014, the Veterinary Diagnostic Laboratory at the University of Minnesota received paraffin-embedded tissue from a 1-week old piglet that had diarrhea submitted by the Instituto Colombiano Agropecuario (ICA) of Colombia. The sample was strongly positive for PEDV by immunohistochemistry. To determine the phylogenetic relationship of the Colombian PEDV strain to global PEDV strains, the total RNA was extracted using MagMAX FFPE total nucleic acid extraction and sequenced using Next Generation Sequencing technology (2, 5, 6). The RNA sample was submitted to the University of Minnesota Genomics Center (UMGC) and was processed using Illumina TruSeq (Illumina, San Diego, CA) library preparation for Illumina MiSeq (Illumina, San Diego, CA). The Illumina paired-end reads (250 nt in length) were trimmed using Trimmomatic software, and duplicate read-pairs were removed (7). Bowtie2 was used to remove swine and bacterial reads (8). The PEDV genome was assembled using the A5 assembly software, and reads were remapped to the PEDV genome to verify the genome assembly (9). The complete genome of strain COL/Cundinamarca/2014 is 28,012 nucleotide (nt) bases in length. The genomic coding regions are found in the following nucleotide ranges: 5′ untranslated region (UTR), nt 1 to 271; replicase polyprotein, nt 272 to 12,625 for 1a, and nt 12,628 to 20,613 for 1b; spike (S), nt 20,613 to 24,770; open reading frame 3 (ORF3), nt 24,773 to 25,444; envelope (E), nt 25,428 to 25,655; membrane (M), nt 25,666 to 26,343; nucleocapsid (N), nt 26,358 to 27,680; and 3′ UTR, nt 27,684 to 28,012. The complete COL/Cundinamarca/2014 PEDV genome is 99.95% similar to the original pandemic strain identified in the United States, Colorado/2013, and 96.73% similar to the classical Belgium strain, CV777. It is most similar to strain USA/Oklahoma418/2014 (99.99%).

Through continued sequencing of PEDV from diverse geographical areas, we hope to keep monitoring and learning about the evolution of PEDV in the Americas. Phylogenetic analysis will help to prevent or mitigate outbreaks of PEDV in the future.

### Nucleotide sequence accession number.

The COL/Cundinamarca/2014 PEDV sequence has been deposited into GenBank under the accession number KU569509.
